# Vicarious Calibration of sUAS Microbolometer Temperature Imagery for Estimation of Radiometric Land Surface Temperature

**DOI:** 10.3390/s17071499

**Published:** 2017-06-26

**Authors:** Alfonso Torres-Rua

**Affiliations:** Utah Water Research Laboratory, Utah State University, Logan, UT 84322, USA; alfonso.torres@usu.edu; Tel.: +1-435-797-0397

**Keywords:** sUAS, vicarious calibration, thermal calibration, surface temperature, atmospheric correction, microbolometer cameras, thermal remote sensing

## Abstract

In recent years, the availability of lightweight microbolometer thermal cameras compatible with small unmanned aerial systems (sUAS) has allowed their use in diverse scientific and management activities that require sub-meter pixel resolution. Nevertheless, as with sensors already used in temperature remote sensing (e.g., Landsat satellites), a radiance atmospheric correction is necessary to estimate land surface temperature. This is because atmospheric conditions at any sUAS flight elevation will have an adverse impact on the image accuracy, derived calculations, and study replicability using the microbolometer technology. This study presents a vicarious calibration methodology (sUAS-specific, time-specific, flight-specific, and sensor-specific) for sUAS temperature imagery traceable back to NIST-standards and current atmospheric correction methods. For this methodology, a three-year data collection campaign with a sUAS called “AggieAir”, developed at Utah State University, was performed for vineyards near Lodi, California, for flights conducted at different times (early morning, Landsat overpass, and mid-afternoon”) and seasonal conditions. From the results of this study, it was found that, despite the spectral response of microbolometer cameras (7.0 to 14.0 μm), it was possible to account for the effects of atmospheric and sUAS operational conditions, regardless of time and weather, to acquire accurate surface temperature data. In addition, it was found that the main atmospheric correction parameters (transmissivity and atmospheric radiance) significantly varied over the course of a day. These parameters fluctuated the most in early morning and partially stabilized in Landsat overpass and in mid-afternoon times. In terms of accuracy, estimated atmospheric correction parameters presented adequate statistics (confidence bounds under ±0.1 for transmissivity and ±1.2 W/m^2^/sr/um for atmospheric radiance, with a range of RMSE below 1.0 W/m^2^/sr/um) for all sUAS flights. Differences in estimated temperatures between original thermal image and the vicarious calibration procedure reported here were estimated from −5 °C to 10 °C for early morning, and from 0 to 20 °C for Landsat overpass and mid-afternoon times.

## 1. Introduction

Spatially distributed estimates of surface temperature can be useful in water resources research for applications in agriculture, geology, riparian habitat, and river corridor analysis [1]. Current efforts to monitor surface temperature using remote-sensing instruments vary in scale from continental (km/pixel) to plant (cm/pixel) and in instrumentation type (satellites, airborne/unmanned sensors) [2] and on-ground infrared radiometer sensors [3,4]. Surface temperature is of value in water resources studies due to its direct impact on processes such as evapotranspiration, soil moisture, open water evaporation, soil/water temperature profiles, climate change, drought monitoring, fish habitat, and others [1,5,6,7,8,9]. Satellite sensors can commonly provide easily accessible temperature information with worldwide coverage. Common satellites with thermal sensors are GOES, MODIS, and Landsat, while others are country-specific solutions, such as CBERS (China-Brazil Earth Resources Satellite) [10]. The imagery provided by these satellites ranges from 30 m/pixel/16 days at its finest resolution (Landsat ETM+/TIRS), to 375 m/pixel/1 day (VIIRS) to 500 m/pixel/1 day (MODIS Terra/Aqua), to 5 km/pixel/1 day (GOES). While the satellite information is used for large-scale surface processes (entire farm to sub-basin and basin scales), the information is of limited value for fine-scale processes that require sub-meter scale measurements and/or multiple measurements on the same day (e.g., sunrise, solar noon, mid-afternoon, night). For these requirements, manned aircraft and sUAS equipped with temperature sensors have been used. Examples of airborne and sUAS thermal applications can be found in [1,11,12,13,14,15,16,17,18,19,20,21,22,23]. Despite its importance in satellite temperature related research, little attention has been paid to atmospheric calibration of thermal imagery from manned aircraft and sUAS. One reason may be the often expensive meteorological sondes (to measures air temperature and relative humidity) that must be used along with atmospheric profile models such MODTRAN and 6S [1,24,25,26,27,28,29,30]. By contrast, the technology implemented in manned aircraft/sUAS (lightweight, relative low-cost) thermal cameras is affected by local weather and flight elevation conditions. Therefore, the absence of standards or recommended procedures for referential calibration and atmospheric correction of thermal cameras for deployment on manned aircraft or sUAS can introduce uncertainty and systematic error in the data they collect and limit its synergistic use in combination with available satellite thermal imagery. The objective of this study was to develop standard procedures for vicarious (sUAS-specific, time-specific, flight-specific, and sensor-specific) atmospheric calibration of thermal cameras used in sUAS platforms.

### 1.1. Microbolometers UAS Temperature Cameras

**Figure 1:**
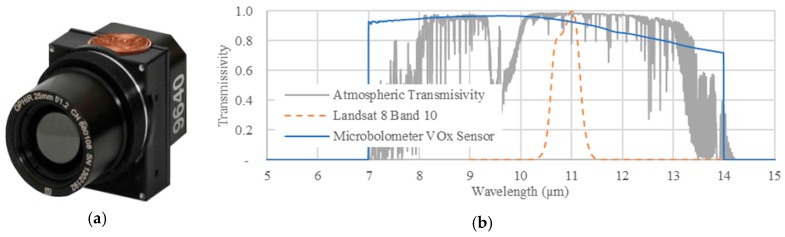
Example of the dimensions of a microbolometer ICI Camera 9640 Series, used in this study (**a**). VOx Spectral Response in the 7 to 14 μm Filter, Landsat 8 Band 10 spectral response and average atmospheric transmissivity in the long infrared region (**b**).

In terms of weight limitations, sUAS (under 25 kg) have only one available radiometric temperature sensor solution: microbolometer infrared sensors (below 200 gr), which have a typical spectral response from ~7 um to ~14 um, using vanadium oxide (VOx) or amorphous silicon (A-Si) as the sensor core [31,32,33]. Figure 1 shows a microbolometer camera that is suitable for thermal remote sensing applications, as well as the spectral response of the sensor. Microbolometer technology uses the responsiveness of the sensor core material to changes in surface temperature that are larger than those of the sensor itself [31]. These sensors are an alternative to the cryogenically cooled thermal technology used in NASA and ESA satellites [34,35,36]. Miniaturized cryogenic temperature sensors exist, but are still too heavy for sUAS (over 4.0 Kg) [37]; thus, they are used mainly for manned aircraft. The manufacturer’s absolute radiometric calibration plays a major role in data quality, with reported laboratory accuracies of ±5 °C (FLIR) [37] and ±°C (ICI). Figure 1 shows the specifications of an ICI microbolometer camera with a typical spectral response [38].

### 1.2. Atmospheric Correction of Surface Temperature

All imaging sensors are affected by atmospheric conditions, as indicated by the atmospheric correction models available for the optical and thermal sensors in satellites such as Landsat, Sentinel-3, MODIS, and others [25,27,39,40,41]. For temperature sensors, the largest sources of distortion are water content in the atmospheric path between the sensor and the surface, in addition to sensor technology and payload integration (i.e., the unit’s accuracy and camera attachment options such as gimbal or frame fitting with/without casing, etc.). For temperature-capable satellites, solutions include an onboard thermal blackbody [27] with a gas-coolant or cryogenic sensor design [42] and minimization of temperature waveband(s) [43] (Figure 1, Landsat 8 spectral response). Nevertheless, these solutions are not available for microbolometer temperature technology (under 200 gr). It is important to compare the spectral response from microbolometer technology for satellites against the atmospheric transmission on the spectral wavebands used to measure surface temperature (7 to 14 um). In this spectral region, water vapor and atmospheric gasses will differentially affect the transmissivity per wavelength and use narrow wavebands: between 8 and 9, and between 10.5 and 12 μm is recommended. However, current microbolometer technology cannot selectively access these recommended narrow wavebands- because the technology itself would decrease the sensing capability of the microbolometer with a narrow band (signal to noise ratio) [38].

For satellites and sUAS, the temperature sensor observes the radiation originally emitted from the surface (L_G_), but reduced or attenuated by atmospheric factors such as the amount of water vapor and other gasses in the atmosphere column between the ground and the sensor, along with weather conditions, sensor view geometry, etc. The measured radiation at the temperature sensor is called “radiance at sensor” (L_S_). The radiation at ground and sensor levels, along with the atmospheric conditions between the sensor and the ground, can be related by using a radiative transfer model [25,44] as presented in Equation (1):

L_S_ = τ ε L_G_ + L_U_ + (1 − ε) L_D_ (1)

where τ is the atmospheric transmissivity, ε is the emissivity of the surface, L_G_ is the radiance of a blackbody target of kinetic temperature T at ground level, L_U_ is the upwelling or atmospheric path radiance, LD is the downwelling or sky radiance, and LS is the radiance measured by the temperature sensor on board the satellite or manned/sUAS. Radiance is in units of W/m^2^/sr/μm, and τ and ε do not have dimensions. If only brightness or radiometric temperature is required, ε can be considered as 1.0, simplifying Equation (1) to Equation (2):

L_S_ = τ L_G_ + L_U_ (2)

For satellite sensors, L_U_ and τ can be determined for the specific image date using a radiative transfer model such as MODTRAN [25,45,46] and 6SV [28,29,30,47] to calculate the scattering and transmission of radiance through the entire earth atmosphere. These models are time-consuming and require input data that is not often available, such as the vertical profile of atmospheric water vapor and other gasses [1]. The quantification of atmospheric water vapor is needed because, while the atmosphere is a mix of gases (nitrogen, oxygen, carbon dioxide, etc.), these can be considered to be present in constant quantities (with resulting constant effect), but water vapor changes continuously in time and space [48]. To determine the radiance of an object from temperature measurements, Planck’s Law allows the nonlinear relationship of the total emittance as a blackbody, at a specific wavelength, to be determined from its temperature and vice versa [49]. When expressed per unit wavelength, the simplified form of Planck’s Law is Equation (3):

W(λ,T) = c_1_(λ^5^(exp(c_2_(λ × T)^−1^) − 1)^−1^ (3)

where W(λ, T) is the total spectral radiant emittance at a temperature per unit area of emitting surface at wavelength (λ) in meters (W·m^−2^·sr^−1^·um^−1^), T is the temperature in Kelvins, c_1_ is 1.1910 × 10^−22^ W·m^−2^·μm^−1^·sr^−1^, and c_2_ is 1.4388 × 10^−2^ m·K. To obtain surface brightness temperature (without emissivity correction) the equation is inverted as follows to Equation (4):

T(λ,W) = c_2_(λ × ln(c_1_(λ^5^W)^−1^) − 1)^−1^ (4)

Equations (3) and (4) use the weighted band center from the specific spectral response of the sensor [1]. It is important to note the linear relationship among the radiance at ground and sensor levels in Equations (1) and (2), while Equations (3) and (4) indicate a nonlinear relationship between temperature and radiance.

## 2. Materials and Methods

### 2.1. Study Site

**Figure 2:**
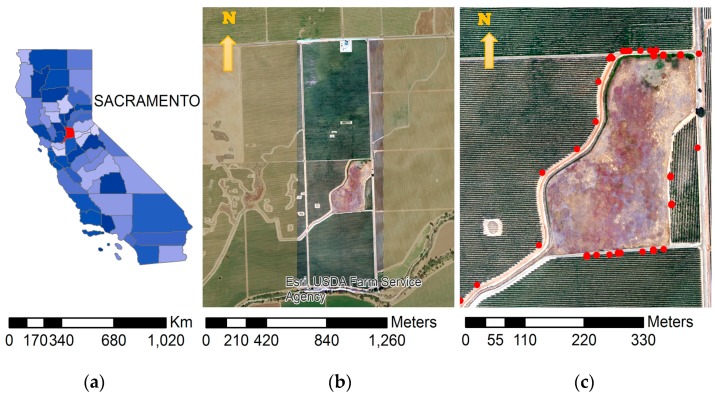
Location of the study area: County location in California (**a**); AggieAir sUAS coverage area in RGB mosaic for all flights (**b**); and close view of sUAS RGB mosaic along with ground temperature sampling locations (dots) (**c**).

The study area is a commercial vineyard near Galt, CA (field center located at 38°17′7.40″ N, 121°6′58.11″ W), operated by E&J Gallo [50]. The farm is equipped with a drip irrigation system and covers an area of approximately 77 hectares (188 acres). Figure 2 shows the location of the farm. The location is also an intensive experimental field by the USDA-ARS Hydrological and Remote Sensing Laboratory—GRAPEX (Grape Remote Sensing Atmospheric Profiling and Evapotranspiration Project).

### 2.2. AggieAir sUAS

**Figure 3:**
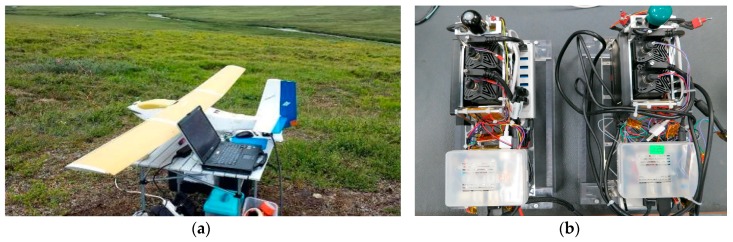
An example of the AggieAir “Minion” sUAS Fixed Wing Aircraft (**a**); and AggieAir custom payload detail (**b**).

The AggieAir sUAS platforms and payloads developed by Utah State University have been widely used for remote sensing assignments in support of research in natural resources, water resources, and agricultural applications. The system incorporates a collection of sUAS remote sensing equipment, including multiple platforms and interchangeable sensor packages. The customizable payload includes short, medium, and long waveband sensors. The extended flight times of AggieAir platforms have incorporated continuous improvements (3.0 h on a single battery charge, up to 12,000 ft MSL, weather sensors, etc.). To achieve scientific accuracy, intensive ground data collection efforts have been conducted to produce reflectance estimation protocols, address camera vignetting, assure accurate image orthorectification, etc. In addition, the optical and thermal cameras are located within a payload frame to minimize atmospheric effects (chilling) on the sensor due to flight elevations (up to 1000 m above ground) and speeds (~50 mph). Figure 3 shows details of the AggieAir “Minion” sUAS and payloads used in this study.

### 2.3. Methods

To develop a vicarious calibration procedure for a microbolometer sensor, the AggieAir sUAS was employed to fly over the area of study and collect thermal imagery during a 3-year campaign (2014–2016) in agricultural lands (vineyards) in California (Figure 2). Temperature information was collected at ground level during each sUAS flight. The flight altitude was 450 m above ground level (AGL) and was constant for all flights. Measurements (and flights) were made at early morning (approximately a half-hour after sunrise), Landsat 8 overpass time (close to solar noon), and mid-afternoon. The AggieAir sUAS navigated over the area of interest based on a pre-programmed flight plan with total flight times of less than 30 min.

The thermal cameras included in this study are described in Table 1. Both microbolometer cameras were acquired from ICI [38]. These instruments were selected partly on the basis of their reported laboratory calibration accuracy and ease of integration with the AggieAir payload [38]. In addition, cameras from this manufacturer have been used by other research groups mentioned in the scientific literature [51,52,53,54]. A National Institute of Standards and Technology NIST traceable temperature camera calibration ambient blackbody was acquired from Palmer Wahl [55]. The “ambient” notation indicates that the blackbody can be used in exterior locations and it does not require cryogenic or external cooling for absolute temperature measurement. Table 1 specifies the technical characteristics of the temperature instruments used in this study.

For this study, the AggieAir sUAS was equipped with visual, near-infrared, and thermal cameras. It was flown over the study area on four different dates and times (early morning, Landsat overpass and mid-afternoon) (Table 2). These flights acquired thermal imagery at 60-cm/pixel resolution at an elevation of 450 m (1476 ft.) AGL for less than 30 min flight time. The three daily flight times were selected to compare sUAS information with specialized algorithms for evapotranspiration alongside Landsat satellite imagery, which are not part of this study. Agisoft Photoscan software [56] was used to create temperature imagery mosaics, while custom MATLAB code and ground control points collected with an RTK GPS system [57] were used to orthorectify the AggieAir imagery [11].

The vicarious calibration methodology used in this study is initially based on the earlier work by [11], which compared georeferenced ground and sUAS temperature pixels for water pools. The present study considered three major vicarious calibration steps as presented in Table 3.

The three main steps of the vicarious calibration methodology (Table 3) are as follows:

• **Before Flight**

Two activities had to be accomplished before the flight: (1) a measurement of the ambient temperature blackbody using the sUAS and ground temperature cameras, and (2) a selection and RTK-GPS survey of the locations to be used for ground data collection during the sUAS flight. The first activity allowed the bias to be determined between the temperature cameras and a NIST-traceable instrument. Given that both instruments include reported accuracies, this activity also allowed the bias source (e.g., instrument or environmental) to be determined [58]. The second activity identified areas of interest in the area of study. In agricultural lands, for example, a range of locations was considered that included bare soil (wet and dry), short vegetation (green, dry), tall canopy, and open water surface. The sub-meter pixel resolution of the sUAS thermal images made it necessary to establish the selected locations with temporary or permanent ground markers and perform GPS surveys with sub-centimeter accuracy, thus the need for RTK-GPS equipment.

• **During Flight**

The previously selected locations were measured with a ground level temperature camera simultaneously with the sUAS flight over the study area. It was important to complete the sUAS flight and the ground data collection in a short amount of time, generally much less than 30 min. This was to avoid the introduction of measurement errors due to diurnal surface temperature changes. A tall, portable frame was erected on a truck to enable a large number of ground temperature images (and pixels) to be collected quickly.

• **After Flight**

After the sUAS and ground temperature data were collected, the sUAS temperature map was developed using mosaicking software (Agisoft Photoscan) and custom MATLAB code to georeference the temperature images from the ground data collection. Temperature pixels were then extracted from both the ground and sUAS images at the resolution of the sUAS image. Temperature pixel data was then transformed into radiance using Equation (3), and the radiometric model (proposed by [25,44]) shown in Equation (2) was applied. Finally, atmospheric transmissivity and atmospheric path radiance were applied to the entire sUAS radiance image (converted from a temperature map) and transformed back into an atmospherically corrected temperature image.

## 3. Results and Discussion

### 3.1. Before Flight

**Figure 4:**
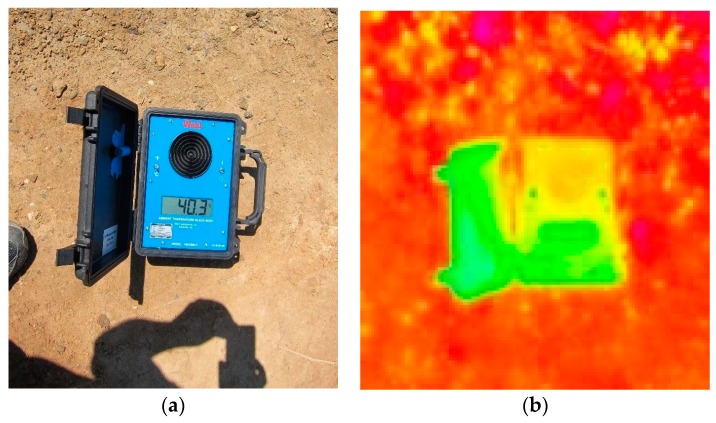
Visual (**a**) and temperature (**b**) images of the NIST traceable ambient temperature blackbody used in this study. Black disk (**a**) is the blackbody temperature sensor.

**Camera–Blackbody Temperature Imagery Measurement:** A total of 213 individual temperature images of the blackbody were compared to determine bias in the microbolometer cameras used in this 3-year study. Figure 4 shows an example of the visual and temperature images of the NIST-traceable ambient temperature blackbody in the field.

**Figure 5:**
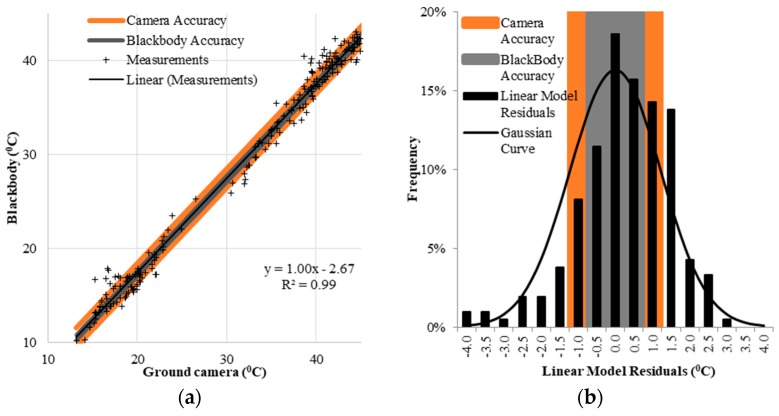
Temperature camera–blackbody comparison for 213 individual measurements. A linear model with a unit slope (1:1) and a constant bias (−2.67 °C) fitted the camera bias value over 3 years of study. Temperature values below 30 °C for the ground camera axis belong to early morning measurements, higher values are for Landsat overpass and mid-afternoon times (**a**); Reported accuracies from blackbody and camera manufacturers can explain up to 48% (gray region) and 68% (orange region) of linear model residuals variability, respectively (**b**).

As specified in Table 1, the temperature blackbody works at an emissivity value of 0.95 ± 0.02, while the microbolometer camera worked at a value of 1.0. Therefore, the blackbody temperature was adjusted to the camera emissivity using the Stefan-Boltzmann Law (5):

T_blackbody·corrected_^4^ = (ε_blacbody_/ε_camera_) × T_blackbody_^4^ (5)

where T_blackbody_ corrected is the emissivity adjusted temperature, ε_camera_ (1.0) and ε_blackbody_ (0.95) are the emissivities of the microbolometer cameras and the blackbody, respectively, and T_blackbody_ is the temperature reported by the ambient blackbody. Once the blackbody temperature was corrected for emissivity, the temperatures (blackbody and cameras) were modeled as shown in Figure 5. In addition, a sensitivity analysis of the camera and blackbody instrument accuracies (Table 1) were performed.

As shown in Figure 5 and Table 4, a strong linear relationship exists between the data from the microbolometer cameras provided by ICI and the ambient blackbody. The linear response to a 1:1-line slope indicates that the ICI microbolometer cameras needed only a constant bias correction expressed by an independent term (−2.67 °C) in the equation shown in Figure 5a. The relationship thus identified was not affected by weather conditions or seasonality (air temperature, wind, humidity, etc.). In addition, Figure 5b shows a residual analysis of the camera-blackbody linear model. The bias residuals have a distribution similar to a Gaussian curve (mean = 0.0 °C, and standard deviation ±1.22 °C). In addition, up to 48% of the residual variability around the mean can be explained by the accuracy of the blackbody (±0.2 °C, ±0.02 ε or ±0.35 °C when both accuracies are combined), and up to 68% of the residual variability around the mean is explained by the accuracy of the thermal camera (±1.00 °C). Therefore, 32% of the variability in the residuals of the modeled bias seemed to be caused by measurement factors (e.g., optical characteristics of the ICI camera such as the point spread function and selection of blackbody pixels from the temperature image).

**Figure 6:**
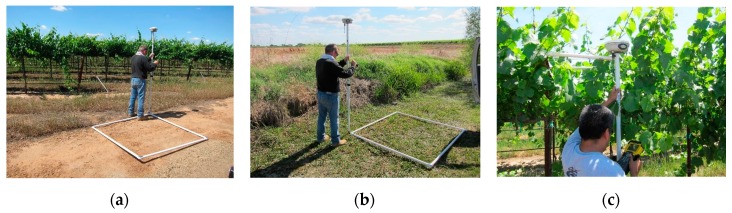
Examples of ground locations for temperature sampling the day before the sUAS flight shown in Figure 2. Locations were delimited using PVC and surveyed using RTK GPS. Note the diversity of locations: bare dry soil (**a**), short green canopy (**b**), and tall canopy (**c**).

**Ground temperature sampling locations:** Different land surfaces were considered during the three-year data collection effort for this project. Examples of different surface types are presented in Figure 2 and Figure 6. These locations were visually homogeneous and covered the range of possible land surfaces in the area of study. An RTK GPS system was used to survey perimeters made with PVC and aluminum tape (1.6 by 1.6 m and 0.8 by 0.8 m) so that sUAS and ground temperature pixels could be accurately located.

### 3.2. During Flight

**Figure 7:**
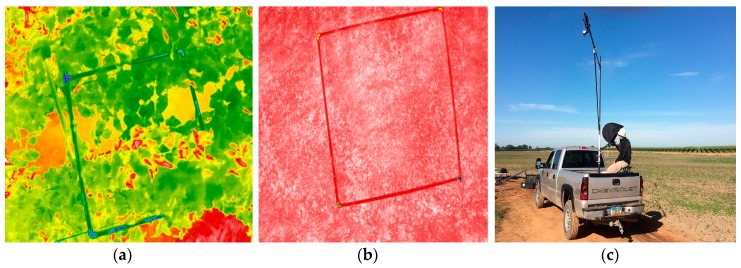
An example of ground temperature samples taken using the temperature camera mounted on a tripod (3 m height) and connected to a laptop loaded with ICI software IR FLASH on 3 May 2016, at Landsat overpass time. Temperature images of the top of vine canopy (**a**) and bare soil (**b**). Temperature camera and tripod mounted over a truck for fast ground temperature sampling (**c**).

**Temperature Ground Sampling:** Simultaneous examples of AggieAir sUAS flights and ground temperature captures are presented in Figure 7:

### 3.3. After Flight

**Figure 8:**
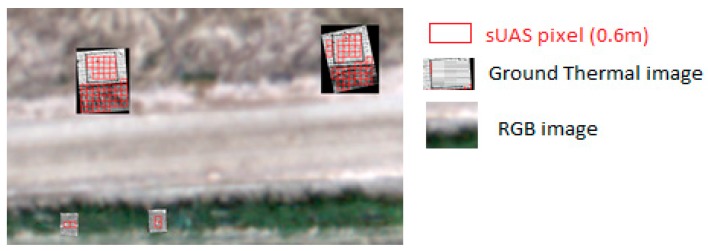
Example of ground temperature images georeferenced from locations shown in Figure 6 and others over a visual image using ArcGIS. The figure showcases thermal temperature images of two bare soil locations (top) and two tall canopies (vines) (bottom). Square grids indicate sUAS and ground pixels that can be extracted for atmospheric radiance calibration.

**Ground and sUAS Pixel Extraction:** After each flight, sUAS temperature maps were developed using data from the sUAS onboard inertial measurement unit (IMU) and GPS receiver. This provided the sUAS location (x, y, z coordinates) and orientation (pitch, roll, yaw) to the Agisoft Photoscan version 1.3 software [56]. RTK-GPS surveyed ground control points, specifically for thermal cameras (aluminum-based blankets), were also included. For the ground temperature camera images, these points were registered using their respective RTK-GPS coordinates and ground PVC frame dimensions using ESRI ArcGIS software. An example of georeferenced ground temperature images is shown in Figure 8.

**Figure 9:**
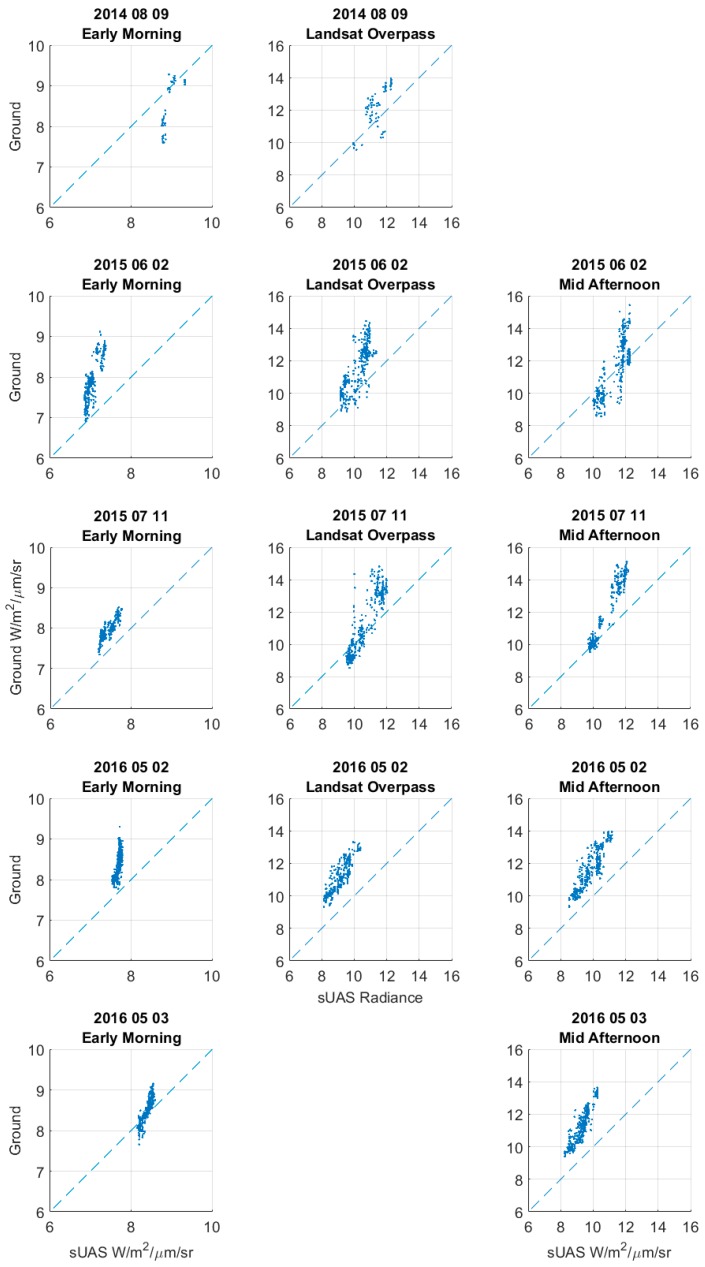
Radiance (W/m^2^/sr/μm) from sUAS (horizontal axis) and ground pixels (vertical axis) for all the sUAS flights in the site of study. The left column is for early morning, a center column for Landsat overpass and the right column for mid-afternoon times respectively.

**Calibration of Atmospheric Radiance Model:** Once extracted, data from the sUAS thermal images and ground temperature values were transformed into radiance Equation (3). The atmospheric radiance model presented in Equation (2) was calibrated for every sUAS flight, and the results of the calibration are shown in Table 5. Figure 9 shows the radiance comparison from sUAS and ground pixels.

In Figure 9 and Table 5 the Equation (2) linear model assumption is confirmed by the linearity of the ground and sUAS pixel comparison. Not all Equation (4) regression calibrations (Table 5) have a high R2 value, due to the scattering of the compared pixels, but small average errors (RMSE) were observed for early morning (<0.5 W/m^2^/μm/sr) and Landsat overpass and mid-afternoon (RMSE < 1 W/m^2^/μm/sr) flights. In all early morning flights, the temperature radiance ranged from 0 to 6 W/m^2^/μm/sr, and for Landsat overpass and mid-afternoon flight times, these ranged from 0 to 16 W/m^2^/μm/sr.

In terms of atmospheric correction parameters, the transmittance (τ) ranged from 0.29 to 0.81 for all flights. The early morning values do not seem to concentrate on a given range (0.29 to 0.81). For Landsat overpass and mid-afternoon, τ values were within the 0.47 to 0.69 range. LU values varied from −0.94 to −5.54 W/m^2^/μm/sr for early morning flights and −2.21 to −5.21 W/m^2^/μm/sr for Landsat overpass and mid-afternoon flights, with no evidence of a preferred value range. The 95% confidence bounds included in Table 5 indicate that even in the best atmospheric conditions (7/11/2015 early morning, τ = 0.81) an atmospheric correction (confidence upper bound = 0.88) is still needed. Furthermore, the confidence bound estimates for LU in Table 5 indicate that there is no record where this parameter can be omitted (zero or positive values). In terms of confidence bound ranges, all τ estimates are within the ±0.1 range and ±1.2 W/m^2^/μm/sr for LU for all measurement times (early morning, Landsat overpass, and mid-afternoon).

**Figure 10:**
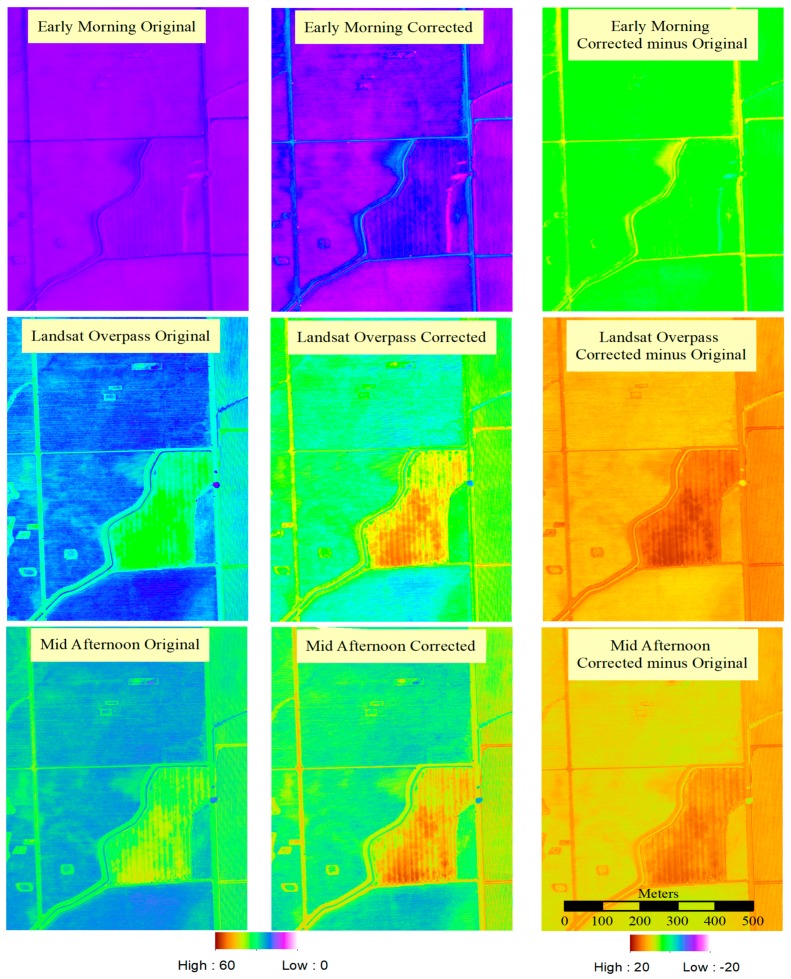
An example of temperature image correction for 2 May 2016 using the vicarious calibration for early morning (**top row**), Landsat overpass (**center row**) and mid-afternoon (**bottom row**). Units in Celsius. The left column is the sUAS original temperature image, the center column is the original sUAS image after the atmospheric radiance correction, and the right column is the difference between the corrected minus original sUAS imagery.

**sUAS Temperature Image Correction:** When the estimation of the atmospheric correction model with the calculation of atmospheric transmissivity τ and radiance LU was completed, the sUAS images were processed by converting them to radiance (W/m^2^/μm/sr) using Equation (3) and then back to temperature using Equation (4). Figure 10 demonstrates the differences in surface temperature due to the atmospheric correction model for a given date. This example shows the sUAS flights for early morning, Landsat overpass and mid-afternoon for 2 May 2016.

The results of the application of τ and L_U_ to a sUAS image, as shown in Figure 10, indicate a significant change in the estimation of the surface temperatures. These changes range from −5 to 10 °C for the early morning images, and 0 to 20 °C for both the Landsat overpass and mid-afternoon images. The significant changes in temperature estimates can be explained by the Stefan-Boltzmann Law, which indicates changes in radiances relate to changes in temperatures at the 4th power, as indicated in Equation (5). Therefore, variations in radiance by the atmospheric radiance correction will translate into significant variation in radiance temperature.

## 4. Conclusions

This study proposes a vicarious calibration methodology for atmospheric correction of microbolometer temperature sensors used on sUAS platforms, such as those of the AggieAir sUAS Research Group at USU. This methodology uses NIST-traceable ambient temperature blackbody and ground level temperature images from different land surfaces from a second temperature camera of the same model and manufacturer. This methodology avoids the use of atmospheric models such as MODTRAN and 6SV, while referring to local measurements during the sUAS flight. The procedure is applicable to any sUAS flight elevation, time, camera spectral response, and camera set up, although the procedure demands additional human effort and surveying equipment for ground data collection. The results of this study indicate that, overall, microbolometer temperature cameras, despite the impact of atmospheric conditions and sUAS setup, can be related to a NIST-traceable temperature device.

The advantage of the proposed vicarious calibration methodology is the accountability of atmospheric and other factors that can affect the acquisition of land surface temperatures, such as daytime and weather conditions, spectral response, camera operational temperature, and others. This methodology requires the use of a microbolometer camera with a laboratory calibration accuracy adequate to the expected posterior analysis. The ICI cameras used in this study have a laboratory accuracy of ±1 °C.

The atmospheric radiance correction of the sUAS thermal imagery requires adequate conversion of the temperature maps from sUAS and georeferenced ground imagery into thermal radiance using the Planck equation and an estimate of the thermal central waveband. The atmospheric radiance calibration provides two main parameters: (1) atmospheric transmissivity (dimensionless), and (2) atmospheric radiance (W/m^2^/μm/sr). These two parameters can be directly applied to the sUAS radiance image. It is important to note that the selection of ground sampling locations plays an important role in the calibration of the atmospheric radiance correction model. It is recommended that different flat locations be considered based on their temperature response (dry/wet, bare, and vegetation-covered soils, top of the canopy for tall crops, open water, snow, etc.), thus creating a temperature gradient necessary for the regression analysis. Shadowed areas are not recommended because sun elevation changes continuously and will affect the temperature conditions of shadow-covered locations during the sUAS flight.

The results from the dates where the vicarious calibration methodology was applied in this study demonstrated that the atmospheric correction model parameters are different for each date and flight time (early morning, Landsat overpass, and mid-afternoon). These results indicate that instantaneous atmospheric conditions (air temperature, water vapor, etc.) between the sUAS and the ground may play a major role in the atmospheric correction parameters values. A simplification of the proposed vicarious calibration methodology might be possible by mounting additional sensors (air temperature, relative humidity, atmospheric pressure, incident radiation, wind speed, etc.) on the sUAS to collect data about air column conditions during periods when the aircraft is climbing or descending. These onboard sensors could build an “atmospheric profile” from the ground to the targeted elevation at the beginning and end of the flight with a continuous monitoring of the weather conditions during the flight. This is an important source of information to support radiometric calibration of thermal imagery for flights that extend for longer time periods (larger than 30 min), or are conducted over time intervals that experience changing atmospheric and/or sunlight conditions. 

A variant on the vicarious calibration presented in this study would be ground temperature collection using a second sUAS, which would carry the ground thermal camera. The expected sUAS elevation should be less than 10 m AGL. Keeping the recommendations about the ground temperature sampling locations as described in this study, the number of pixels available for the radiance atmospheric model can increase by an order of magnitude while keeping the necessary sUAS flight time as described in the procedure presented here.

Future work involves the comparison and cross-calibration of other temperature sensors and image sources, such as atmospherically corrected Landsat, temperature canopy sensors, and hemispherical radiometers towards the integration of multiple thermal measurements and emissivity estimation.

## Figures and Tables

**Table 1:** Instruments used to collect temperature information in this study.

Instrument	Blackbody	2014	2015–2016
Brand/Model	Wahl Palmer/WD1042	ICI/7640-P	ICI/9640-P
Weight (gr)	1000	148	141
Image Size (pixel)	--	640 by 480	640 by 480
Spectral Range (μm)	--	7 to 14	7 to 14
Spectral Band Centre (μm)	--	10.35	10.35
Operating Range	−40 to 70 °C	−20 to 100 °C	−40 to 140 °C
Reported Accuracy	±0.2 °C	±1.0 °C or ± 1.0%	±1.0 °C
Reported Emissivity	0.95 ± 0.02	1.0	1.0
NIST Traceable?	YES	NOT REPORTED	NOT REPORTED

**Table 2:** AggieAir sUAS flights included in this study (Times in Pacific Daylight Time zone).

Date	Early Morning Flights		Landsat Overpass Flights		Mid-Afternoon Flights	
	Launch	Landing	Launch	Landing	Launch	Landing
09 August 2014	7:10 AM	7:30 AM	11:30 AM	11:50 AM	No UAS flight	
02 June 2015	6:51 AM	7:32 AM	11:21 AM	12:06 PM	2:54 PM	3:20 PM
11 July 2015	6:37AM	7:11 AM	11:26 AM	12:00 PM	2:58 PM	3:31 PM
02 May 2016	8:13 AM	8:35 AM	12:53 PM	1:17 PM	3:52 PM	4:16 PM
03 May 2016	8:40 AM	9:06 AM	No UAS flight		1:35 PM	2:00 PM

**Table 3:** Followed vicarious calibration methodology used in this study.

Steps	Activity Description
Before Flight	• Camera—blackbody temperature measurement • GPS survey of ground temperature sampling locations
During Flight	• Temperature ground sampling
After Flight	• Ground/sUAS temperature pixel extraction • Calibration of Radiative Transfer Model • sUAS temperature image correction

**Table 4:** Statistics for Temperature camera—blackbody linear model analysis.

Model	Slope (95% Confidence Bounds)	Bias (95% Confidence Bounds)	R^2^	RMSE (C°)	Reported Camera Accuracy (C°)	Reported Blackbody Accuracy (C°)
Linear	1.00 (0.99 1.02)	−2.67 (−3.19–2.22)	0.99	1.23	±1.00	±0.35

**Table 5:** Statistical results from atmospheric radiance model (τ and Lu) using ground and sUAS pixels.

Date	Flight Time	τ (95% Confidence Bounds)	L_u_ (95% Confidence Bounds) W/m^2^/μm/sr	r^2^	RMSE W/m^2^/μm/sr	Used Pixels
8/9/2014	Early Morning	0.40 (0.51 0.33)	−5.54 (−9.38–3.03)	0.65	0.37	48
	Landsat Overpass	0.69 (0.88 0.57)	−2.94 (−6.75–0.45)	0.58	0.84	63
6/2/2015	Early Morning	0.35 (0.38 0.33)	−4.28 (−5.10–3.56)	0.71	0.26	336
	Landsat Overpass	0.57 (0.62 0.53)	−3.61 (−4.88–2.53)	0.62	0.88	330
	Mid Afternoon	0.53 (0.58 0.50)	−5.17 (−6.49–4.04)	0.68	0.97	330
7/11/2015	Early Morning	0.81 (0.88 0.76)	−0.94 (−1.58–0.39)	0.73	0.12	283
	Landsat Overpass	0.49 (0.52 0.47)	−5.08 (−5.86–4.38)	0.83	0.77	352
	Mid Afternoon	0.47 (0.48 0.45)	−5.21 (−5.71–4.74)	0.94	0.47	278
5/2/2016	Early Morning	0.29 (0.33 0.27)	−5.23 (−6.66–4.05)	0.53	0.21	343
	Landsat Overpass	0.62 (0.63 0.60)	−2.21 (−2.49–1.94)	0.95	0.46	299
	Mid Afternoon	0.65 (0.69 0.62)	−2.25 (−2.94–1.62)	0.79	0.55	299
5/3/2016	Early Morning	0.43 (0.45 0.41)	−4.74 (−5.49–4.07)	0.8	0.13	326
	Mid Afternoon	0.52 (0.55 0.50)	−3.38 (−4.01–2.82)	0.83	0.43	349
